# Increased risk of asthma in female night shift workers

**DOI:** 10.1183/23120541.00137-2025

**Published:** 2025-11-17

**Authors:** Robert J. Maidstone, David W. Ray, Junxi Liu, Jack Bowden, Martin K. Rutter, Hannah J. Durrington

**Affiliations:** 1 Division of Immunology, Immunity to Infection and Respiratory Medicine, School of Biological Sciences, Faculty of Biology, Medicine and Health, University of Manchester, Manchester, UK; 2 National Institute for Health and Care Research (NIHR) Oxford Health Biomedical Research Centre, John Radcliffe Hospital, Oxford, UK; 3 NIHR Oxford Biomedical Research Centre, John Radcliffe Hospital, Oxford, UK; 4 Oxford Centre for Diabetes, Endocrinology and Metabolism, University of Oxford, Oxford, UK; 5 Oxford Kavli Centre for NanoScience Discovery, University of Oxford, Oxford, UK; 6 Nuffield Department of Population Health, Oxford Population Health, University of Oxford, Oxford, UK; 7 Department of Clinical and Biomedical Sciences, Faculty of Health and Life Sciences, University of Exeter, Exeter, UK; 8 Division of Diabetes, Endocrinology and Gastroenterology, School of Medical Sciences, Faculty of Biology, Medicine and Health, University of Manchester, Manchester, UK; 9 Manchester Diabetes Centre, Manchester University NHS Foundation Trust, Manchester Academic Health Science Centre, Manchester, UK; 10 Wythenshawe Hospital, Manchester University NHS Foundation Trust, Wythenshawe, Manchester, UK; 11 These authors contributed equally

## Abstract

**Rationale:**

Asthma is more common in females and more common in night shift workers. Since increasing numbers of females are becoming shift workers, it is important to determine if the risk of shift work-associated asthma is higher in females. The objective of the present study was to determine if increasing frequency of shift work is more strongly related to prevalent asthma in females than in males.

**Method:**

We used cross-sectional data from >280 000 UK Biobank participants and logistic regression models adjusted for demographic and lifestyle factors to describe sex differences in prevalent asthma phenotypes related to shift work frequency. To obtain mechanistic insights, we explored associations with chronotype, sex hormones and menopause.

**Results:**

Female permanent night shift workers had higher covariate-adjusted odds of moderate–severe asthma (odds ratio (OR) 1.50, 95% confidence interval (CI) 1.18–1.91) than female dayworkers, but there was no corresponding relationship among males (OR 0.95, 95% CI 0.72–1.26; sex interaction p-value=0.01). Similar relationships were observed for “all asthma” and for “wheeze or whistling in the chest”. Female shift work-related asthma was driven by relationships in postmenopausal women not using hormone replacement therapy (HRT) (adjusted OR 1.89 (95% CI 1.24–2.87) for moderate–severe asthma; sex interaction p-value=0.02 in permanent night shift workers, compared with dayworkers), but these relationships attenuated to the null in postmenopausal women using HRT.

**Conclusion:**

Our finding that increasing shift work frequency is more strongly related to asthma in females than in males could have public health implications. Intervention studies should determine if modifying shift work schedules or using HRT can reduce asthma risk in females.

## Shareable abstract

**Increasing shift work frequency is more strongly related to asthma in females than in males** https://bit.ly/4jfPml8

## Introduction

Asthma is a highly rhythmic inflammatory disease, with symptoms worsening overnight [1], coinciding with exaggerated airway narrowing and increased airway inflammation [2].

Using data from >280 000 UK Biobank participants [3], we previously showed that “permanent” night shift workers had a higher likelihood of moderate–severe asthma than dayworkers, possibly due to disrupted circadian rhythms.

Asthma predominantly affects females, who have more severe disease and higher hospitalisation and death rates than males [4]. The higher asthma prevalence among females starts at puberty and is associated with fluctuations in hormones during menstruation, pregnancy and menopause, suggesting a possible causal role for female sex hormones in asthma pathogenesis [5].

Oestrogens regulate the circadian molecular clock [6], and oestrogen receptor β is expressed in a circadian manner, particularly in lung epithelial cells, thereby coupling the circadian phase to oestrogen action in the lung [7]. Females tend to have an earlier chronotype (preference for morningness) than males, which is probably related to having a shorter intrinsic circadian period [8–10].

Night shift work is currently undertaken by ∼20% of workers [11] and, of the recent increase in shift worker numbers, two thirds are female [12]. Therefore, it is important to understand if shift work is linked to a higher risk for asthma in females than in males.

Using data on UK Biobank participants, our main aim was to investigate sex differences in the association between shift work and asthma. To obtain mechanistic insights, we explored relationships of chronotype and sex hormones with asthma, and how asthma risk in female shift workers is related to menopausal status.

## Methods

UK Biobank recruited 502 540 participants (5% of those invited) aged 40–69 years [13]. Our analysis was restricted to participants in paid employment or who were self-employed at baseline (n=274 541) [14].

### Cases of asthma

Cases of asthma and moderate–severe asthma were defined using a combination of self-reported doctor diagnosis and medication use, excluding those with contradictory information [15] (supplementary figure 1A). We identified 13 589 (5.3% of our study cohort) asthma cases, of which 4549 (1.9%) had moderate–severe asthma.

We also analysed data from participants who answered positively to the question, “Have you ever experienced wheeze or whistling in the chest within the last year?” and those participants with obstructive pulmonary function tests (<80% of the forced expiratory volume in 1 s (FEV_1_ ) % predicted). We focused our further analyses on individuals with moderate–severe asthma as this was most likely to capture individuals with current, active asthma.

### Shift work and chronotype

We categorised current shift work status as 1) “dayworker”, 2) “shift worker, but only rarely if ever nights”, 3) “irregular shift work including nights” and 4) “permanent night shifts”, based on responses to the UK Biobank baseline questionnaire [15]. Similarly, self-reported chronotype was categorised as 1) “definitely a ‘morning’ person”, 2) “intermediate chronotype” and 3) “definitely an ‘evening’ person” [15].

### Sex hormones

Testosterone, sex hormone-binding globulin (SHBG) and oestradiol were measured using RIQAS Chemiluminescent Immunoassay on Beckman Coulter UniCel DxI 800 [16]. Oestradiol measurements below the reportable range (175 pmol·L^−1^) were considered the referent group. In the continuous analysis, measurements below the reportable range were set to the cut-off value.

### Statistical analysis

Multivariable-adjusted logistic regression models estimated odds ratios for the presence of asthma phenotypes associated with increasingly frequent shift work categories using dayworkers as the referent group.

We used three models, as in our prior study [15] (supplementary figure 1B), including the following covariates: model 1 adjusted for age only; model 2 adjusted for age, ethnicity, chronotype, Townsend Deprivation Index (TDI, a measure of material deprivation), days exercised, alcohol status (current, previous or never) and alcohol weekly intake, length of working week and whether the current job is considered to have an occupational asthma risk or requires a medical examination; model 3 adjusted for model 2 covariates plus potential mediators of the shift work–asthma relationship (confirmed through mediation analysis; see supplementary table 1): body mass index (BMI), smoking history, pack-years smoked, sleep medication and sleep duration [17–22]. Participants with missing covariate data were excluded from analyses.

Interaction analyses were performed by a likelihood ratio test between models with and those without interaction terms.

In all statistical tests, p<0.05 was considered statistically significant. All analyses were performed using R (v.4.0.2) [23] within Rstudio (v.2024.04.2–764) [24], plotted using ggplot2 (v.3.5.1) [25] and patchwork (v1.2.0) [26].

## Results

### Population characteristics

Demographic details by sex and shift work status are shown in table 1. Male and female workers were similarly aged; females tended to be less likely to smoke than males. Female workers tended to drink less alcohol than males. Both sexes showed similar trends across the work schedule groups. The ethnicity of participants was similar between the sexes. Female workers, especially those working nights, tended to work fewer weekly hours than males and come from more deprived areas.

**TABLE 1:** Social-demographic characteristics by current shift work exposure (n=274 541)

	Sex	Current work schedule			
		Dayworkers	Shift work, but never or rarely night shifts	Irregular shift work including nights	Permanent night shift work
**n**	F	120 638	12 182	6429	2608
	M	106 418	11 201	10 833	4232
**Age, years**	F	52.92±6.80	52.79±6.83	51.43±6.56	52.38±6.80
	M	53.98±7.37	53.23±7.26	51.80±6.98	51.79±6.90
**Smoker, %**					
Never	F	61.12	56.63	57.54	56.33
	M	54.73	50.25	49.89	49.03
Previous	F	30.04	30.21	27.16	25.88
	M	34.01	34.37	32.54	32.94
Current	F	8.60	12.81	14.95	17.56
	M	11.04	15.04	17.07	17.70
**Smoking pack-years**	F	5.10±10.75	6.99±12.92	6.99±12.98	8.52±14.60
	M	7.83±15.03	10.25±17.58	10.80±17.70	11.63±18.63
**Drink alcohol daily, %**	F	16.13	13.11	12.02	7.17
	M	25.2	20.78	18.30	12.17
**Monthly units of alcohol consumed^#^**	F	35.80±45.45	31.15±46.12	30.77±47.73	23.61±41.07
	M	82.06±89.02	78.93±101.45	82.81±100.99	71.70±87.70
**Sleep duration, hours**	F	7.10±1.07	7.01±1.26	6.93±1.31	6.75±1.62
	M	6.99±0.96	6.89±1.15	6.81±1.21	6.64±1.38
**Chronotype, %**					
Morning	F	24.00	25.78	24.04	21.78
	M	22.63	25.63	22.26	17.83
Intermediate	F	60.02	57.97	56.39	51.53
	M	57.16	54.45	54.51	48.44
Evening	F	7.94	7.94	10.85	16.95
	M	8.06	7.60	9.27	16.81
Do not know	F	7.93	8.01	8.45	9.24
	M	11.99	12.01	13.67	16.31
**Ethnicity, %**					
White British	F	88.29	83.66	78.50	77.80
	M	89.09	83.46	81.29	83.48
White Other	F	6.71	7.35	7.78	6.86
	M	6.01	6.62	6.52	5.46
Mixed	F	0.77	0.95	1.35	1.07
	M	0.50	0.79	0.73	0.61
Asian	F	1.40	2.72	2.47	2.68
	M	2.01	4.49	4.46	3.80
Black	F	1.54	2.77	6.16	7.02
	M	1.17	2.48	4.00	4.30
Chinese	F	0.37	0.56	0.68	1.04
	M	0.28	0.36	0.30	0.45
Other	F	6.71	7.35	7.78	6.86
	M	6.01	6.62	6.52	5.46
**Weekly work hours**	F	30.84±12.15	31.40±11.73	34.98±12.57	34.73±14.08
	M	39.16±11.59	40.28±11.34	43.55±12.45	43.32±11.49
**Single occupancy, %**	F	16.94	20.06	21.61	20.84
	M	13.97	17.24	16.75	16.71
**Urban area, %**	F	86.23	89.06	88.84	90.17
	M	85.43	89.94	89.47	91.35
**Townsend Deprivation Index, median (IQR)**	F	−2.17 (−3.65 to 0.28)	−1.41 (−3.22 to 1.53)	−1.23 (−3.15 to 1.75)	−0.98 (−3.08 to 2.08)
	M	−2.34 (−3.76 to 0.04)	−1.26 (−3.17 to 1.64)	−1.30 (−3.20 to 1.78)	−1.09 (−3.01 to 2.00)

Data are presented as mean±sd unless otherwise stated. F: female; M: male; IQR: interquartile range. ^#^: Indicates variable with >20% missing data from participants.

Health characteristics are presented in table 2. Female and male workers showed similar trends in BMI, with increasing BMI associated with greater frequency of night shift work, especially among females.

**TABLE 2:** Health characteristics by current shift work exposure (n=274 541)

	Sex		Current work schedule		
		Dayworkers	Shift work, but never or rarely night shifts	Irregular shift work including nights	Permanent night shift work
**n**	F	120 638	12 182	6429	2608
	M	106 418	11 201	10 833	4232
**BMI, kg·m^–2^**	F	26.64±5.05	27.42±5.40	27.69±5.59	28.47±5.78
	M	27.61±4.06	28.20±4.42	28.51±4.39	28.54±4.20
**Birthweight, kg**	F	3.25±0.61	3.23±0.66	3.24±0.64	3.22±0.69
	M	3.45±0.65	3.42±0.69	3.44±0.69	3.38±0.72
**High cholesterol, %**	F	5.36	6.23	6.18	7.67
	M	10.79	11.05	10.00	10.56
**Sleep apnoea, %**	F	0.11	0.10	0.20	0.04
	M	0.48	0.55	0.54	0.40
**COPD**	F	0.15	0.21	0.19	0.19
	M	0.12	0.21	0.19	0.09
**Emphysema/chronic bronchitis**	F	0.64	0.97	1.06	0.88
	M	0.76	1.20	1.16	1.28
**Bronchiectasis, %**	F	0.15	0.16	0.06	0.15
	M	0.12	0.09	0.01	0.12
**Interstitial lung disease, %**	F	0.01	0.01	0.00	0.00
	M	0.03	0.01	0.05	0.02
**Other respiratory problems, %**	F	0.11	0.13	0.14	0.12
	M	0.12	0.21	0.16	0.05
**Gastro-oesophageal reflux, %**	F	2.83	3.44	3.42	4.26
	M	3.63	3.96	4.17	4.23
**Type 1 diabetes, %**	F	0.06	0.07	0.03	0.08
	M	0.08	0.05	0.05	0.05
**Type 2 diabetes, %**	F	0.28	0.42	0.39	0.42
	M	0.57	0.77	0.71	0.66
**Hypertension, %**	F	17.21	19.14	18.34	20.28
	M	23.80	25.60	24.24	25.17
**Cardiovascular disease, %**	F	2.20	2.94	2.80	2.91
	M	5.65	5.93	5.21	4.87
**Depression, %**	F	5.97	7.31	7.11	7.36
	M	3.13	3.52	3.07	3.26
**Anxiety/panic attacks, %**	F	1.39	1.53	1.28	1.30
	M	0.89	0.95	0.66	0.76
**Hay fever/allergic rhinitis/allergy to house dust mite, %**	F	6.31	5.82	5.62	5.21
	M	6.83	5.76	5.86	4.96
**Eczema/dermatitis, %**	F	2.75	2.35	2.77	2.34
	M	2.69	2.42	2.10	2.08
**Allergy or anaphylactic reaction to food/drug, %**	F	1.57	1.48	1.62	1.73
	M	0.88	0.82	0.81	0.73
**Testosterone, nmol·L^−1^**	F	1.15±0.68	1.14±0.68	1.16±0.67	1.19±1.05
	M	12.06±3.62	12.11±3.79	12.10±3.65	12.31±3.82
**Oestradiol, pmol·L^−1^**	F	551.72±487.86	539.76±425.59	541.74±453.25	551.5±448.08
	M	226.06±88.75	225.06±70.61	223.48±79.49	229.42±77.60
**SHBG, nmol·L^−1^**	F	63.76±31.78	62.00±32.50	61.61±31.80	62.11±32.24
	M	37.44±15.59	36.91±16.11	35.99±15.31	36.51±15.83
**Free Androgen Index**	F	2.34±2.30	2.43±2.37	2.48±2.89	2.51±2.93
	M	35.57±14.24	36.56±19.71	37.40±19.28	37.70±17.35
**Free testosterone, nmol·L^−1^**	F	0.02±0.01	0.02±0.01	0.02±0.01	0.02±0.02
	M	0.23±0.07	0.23±0.07	0.23±0.07	0.24±0.07

Data are presented as mean±sd unless otherwise stated. F: female; M: male; BMI: body mass index; SHBG: sex hormone-binding globulin.

### Female shift workers have higher odds of moderate–severe asthma

We first investigated sex differences in the association between increasing frequency of night shift work and prevalent asthma. After adjusting for age (model 1), female shift workers had higher odds of moderate–severe asthma than female dayworkers (supplementary table 2: shift work, but never or rarely night shifts, odds ratio (OR) 1.16 (95% confidence interval (CI) 1.02–1.32); irregular shift work including nights, OR 1.18 (95% CI 0.99–1.40); and permanent night shift work, OR 1.54 (95% CI 1.22–1.95)). In contrast, male shift workers did not have a higher likelihood of moderate–severe asthma than male dayworkers (shift work, but never or rarely night shifts, OR 1.05 (95% CI 0.89–1.23); irregular shift work including nights, OR 0.94 (95% CI 0.79–1.13); and permanent night shift work, OR 0.91 (95% CI 0.69–1.20)).

**FIGURE 1:**
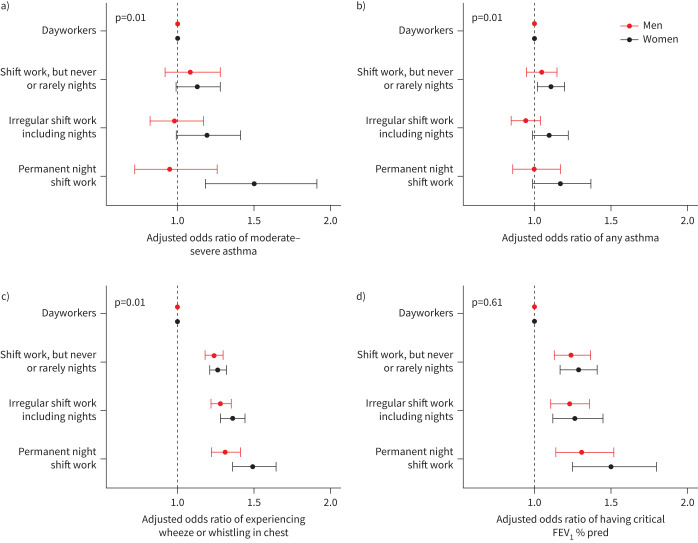
Adjusted odds ratios (95% confidence intervals) of asthma and asthma symptoms by current shift work exposure, stratified by sex. Odds ratios from model 2 of a) moderate–severe asthma (n=245 356; 117 350 male and 128 006 female), b) any asthma (n=254 396; 121 387 male and 133 009 female), c) experiencing wheeze or whistling in chest within the last year (n=268 255; 127 374 male and 140 881 female) and d) having a critical (<80%) forced expiratory volume in 1 s (FEV_1_ ) % predicted (n=87 358; 39 061 male and 48 297 female). Adjusted for covariates in model 2: age, ethnicity, Townsend Deprivation Index, alcohol status, daily alcohol intake, days exercised (walked, moderate and vigorous), length of working week, job asthma risk, job medical required and chronotype.

Some of these relationships were maintained, but were attenuated, after covariate adjustment (figure 1, supplementary table 2; model 2, *e.g.* female permanent night shift workers, OR 1.50, 95% CI 1.18–1.91) and after further adjustment for potential moderators (sleep duration, smoking and BMI; supplementary table 2; model 3, *e.g.* female permanent night shift workers, OR 1.30, 95% CI 1.02–1.66). An interaction between sex and the frequency of night shift work was found across all models, indicating that increasing frequency of night shift work was more strongly related to a higher likelihood of prevalent moderate–severe asthma in females than in males (p=0.01).

We primarily present results from model 2, which is likely to provide the best estimate of the direct effect of shift work on asthma. These data are presented in the figures, and our full results for models 1 and 3 can be found in the supplementary tables.

Female shift workers also had higher odds of any asthma than female dayworkers (after adjusting for model 2 covariates: shift work, but never or rarely night shifts, OR 1.11 (95% CI 1.02–1.20); irregular shift work including nights, OR 1.10 (95% CI 0.99–1.22); and permanent night shift work, OR 1.17 (95% CI 0.99–1.37)), whereas male shift workers showed no such relationships (shift work, but never or rarely night shifts, OR 1.05 (95% CI 0.95–1.15); irregular shift work including nights, OR 0.94 (95% CI 0.85–1.04); and permanent night shift work, OR 1.00 (95% CI 0.86–1.17); supplementary table 3). We found evidence of an interaction between sex and shift work status indicating that increasing frequency of night shift work was more strongly related to the presence of “any asthma” in females than in males (p=0.01, figure 1b). The significant sex–shift work interaction persisted in models adjusted for age (model 1) and after adjusting for potential moderators (model 3) (supplementary table 3).

Both female and male shift workers had a higher risk of experiencing wheeze or whistling in the chest (within the last year) than dayworkers; see figure 1c (*e.g.* model 2: permanent female night shift workers, OR 1.49 (95% CI 1.36–1.64); male permanent night shift workers, OR 1.31 (95% CI 1.22–1.41)). We found evidence of a sex–shift work interaction (p=0.01; model 2). The increased odds among male and female shift workers over dayworkers persisted in models 1 and 3; however, a significant sex–shift work interaction was found only in models 1 and 2 (supplementary table 4).

Furthermore, female and male shift workers also had a higher risk of having obstructed lung function than corresponding female and male dayworkers; see figure 1d (*e.g.* model 2: permanent female night shift workers, OR 1.50 (95% CI 1.25–1.80); male permanent night shift workers, OR 1.31 (95% CI 1.14–1.52)). The higher odds among male and female shift workers over dayworkers persisted in model 1. However, they were lost for all shift work schedules bar “shift work, but never or rarely nights”, for which they attenuated, after the addition of potential moderators (model 3). No sex–shift work interaction was found in any of the models (supplementary table 5).

Using data on employment history, females showed a higher risk of moderate–severe asthma associated both with higher monthly number of night shifts (OR 1.39 (95% CI 1.04–1.88) for >10 night shifts per month; model 2, supplementary table 6) and with higher lifetime duration of night shifts (OR 1.28 (95% CI 1.03–1.59) for >10 years of night shifts; model 2, supplementary table 7), but a significant trend was found only among females for lifetime duration (p=0.02).

### Role of chronotype and sex hormones in the sex-specific relationships linking shift work with prevalent asthma

**FIGURE 2:**
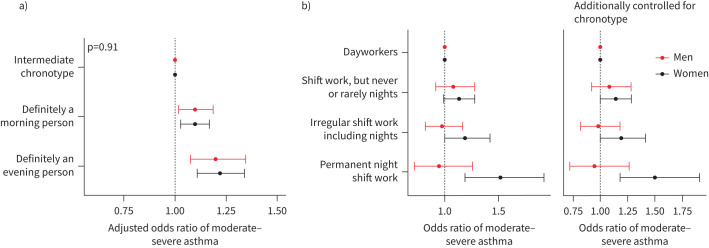
a) Adjusted odds ratios (95% confidence intervals (CIs)) of moderate–severe asthma by chronotype, stratified by sex (n=377 515; 165 629 male and 211 886 female, model 2). b) Adjusted odds ratios (95% CIs) of moderate–severe asthma by current shift work exposure, stratified by sex. Odds ratios of moderate–severe asthma from model without chronotype (left) and model including chronotype (right) (n=245 356; 117 350 male and 128 006 female, model 2). Model 2 covariates: age, ethnicity, Townsend Deprivation Index, alcohol status, daily alcohol intake, days exercised (walked, moderate and vigorous), length of working week, job asthma risk, job medical required.

We previously showed that moderate–severe asthma is linked to extreme chronotype [13]. These associations remained in a sex-stratified analysis (figure 2a), with no evidence of interaction between sex and chronotype (supplementary table 8). Inclusion of chronotype in sex-stratified models of shift work–asthma relationships had negligible effects, including no change in the interaction between sex and shift work on moderate–severe asthma (figure 2b and supplementary table 9).

**FIGURE 3:**
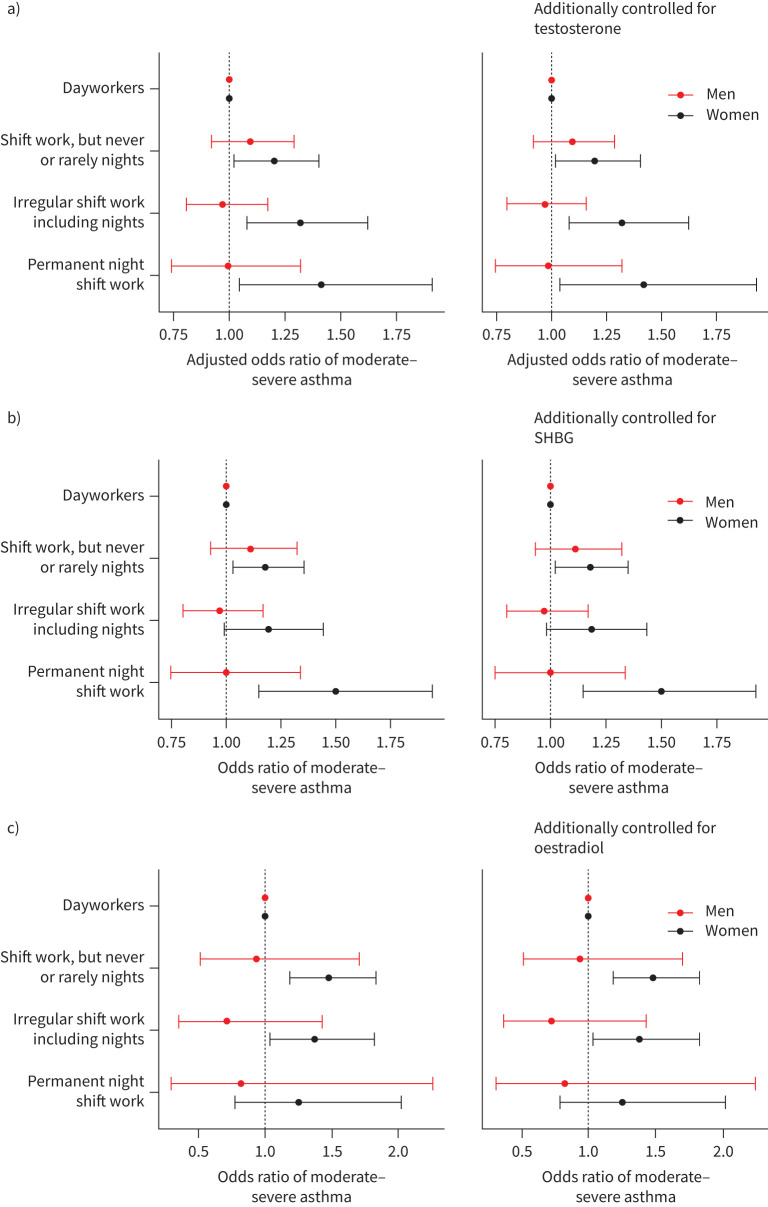
Adjusted odds ratios (95% confidence intervals) of moderate–severe asthma by current shift work exposure, stratified by sex. Odds ratios of moderate–severe asthma from model 2 (left) and model 2 plus additionally controlled for an additional covariate (right). Covariates additionally controlled for: a) testosterone (n=216 364), b) sex hormone-binding globulin (SHBG) (n=211 866) and c) oestradiol (n=49 540). Model 2 covariates: age, ethnicity, Townsend Deprivation Index, alcohol status, daily alcohol intake, days exercised (walked, moderate and vigorous), length of working week, job asthma risk, job medical required and chronotype.

Higher levels of testosterone and SHBG were associated with lower prevalences of moderate–severe asthma in both females and males (supplementary figure 2a,b and supplementary tables 10 and 11). Corresponding relationships with oestradiol were null (supplementary figure 2C and supplementary table 12). Adding testosterone, SHBG or oestradiol as covariates in sex-stratified models of shift work–asthma relationships had negligible effects (figure 3a–c and supplementary tables 13–15).

Given the age of UK Biobank participants (40–70 years) and the paucity of oestradiol data (83% of female participants had “out of range” results (<175 pmol·L^−1^), we next investigated the influence of the menopause on asthma risk.

### Menopause and asthma

**FIGURE 4:**
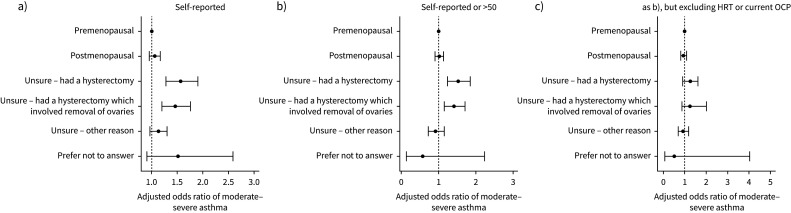
Adjusted odds ratios (95% confidence intervals) of moderate–severe asthma by menopause status: a) self-reported, b) self-reported or aged >50 and c) self-reported or aged >50, but excluding females using exogenous sex hormones, such as the oral contraceptive pill (OCP) or hormone replacement therapy (HRT).

When compared with premenopausal females, postmenopausal females had a similar likelihood of having moderate–severe asthma (figure 4a and supplementary table 16). However, females who were unsure of their menopausal status and reported having had a hysterectomy, with or without an oophorectomy, had 1.4–1.5-fold higher adjusted odds of moderate–severe asthma than premenopausal females (supplementary table 16). We saw similar results when defining postmenopausal status to additionally include females aged >50 (figure 4b and supplementary table 17), but results attenuated to the null after excluding females using exogenous sex hormones, such as the oral contraceptive pill (OCP) or hormone replacement therapy (HRT) (figure 4c and supplementary table 18).

As a sensitivity analysis, we reran the shift work moderate–severe asthma analysis in females who had not had a hysterectomy (supplementary table 19); the results were similar to those observed in the complete female cohort (figure 1a).

### Role of menopause in the relationship between shift work and prevalent asthma

**FIGURE 5:**
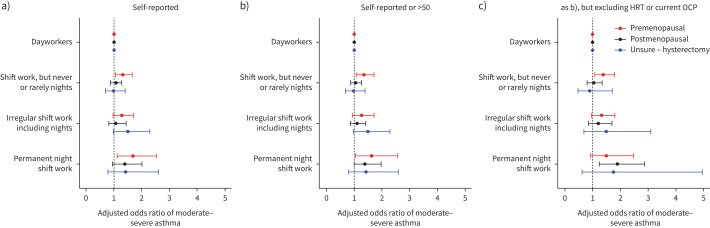
Adjusted odds ratios (95% confidence intervals) of moderate–severe asthma by current shift work exposure, stratified by menopause status: a) self-reported, b) self-reported or aged >50 and c) self-reported or aged >50, but excluding females using exogenous sex hormones, such as the oral contraceptive pill (OCP) or hormone replacement therapy (HRT).

Premenopausal permanent night shift workers and premenopausal females who worked “shift work, but never or rarely nights” had higher likelihoods of moderate–severe asthma than premenopausal dayworkers (figure 5a), but these relationships attenuated to the null after adjusting for model 3 covariates (supplementary table 2). No relationships were found in postmenopausal/hysterectomy groups. We found no interactions between menopause status/hysterectomy and the shift work–asthma relationship in any of the models (supplementary tables 20 and 21).

After excluding participants receiving HRT/OCP, premenopausal shift workers who never or rarely worked nights had a higher likelihood of moderate–severe asthma, but these relationships attenuated to the null after adjusting for model 3 covariates (figure 4c and supplementary table 22). After excluding participants receiving HRT/OCP, postmenopausal permanent night shift workers had 1.9-fold higher odds of moderate–severe asthma than dayworkers; this was robust to covariate adjustment. We found no evidence of an interaction between menopause/hysterectomy status overall in the shift work–asthma relationship; however, numbers were small in these subgroups (supplementary table 22).

To determine whether the sex effect on the shift work-related asthma relationship was driven by menopausal status, we tested for a sex–shift work interaction. An interaction between sex and shift work frequency was found between males and premenopausal, but not postmenopausal, females (supplementary table 23). However, after excluding females receiving HRT/OCP, an interaction between sex and night shift work frequency was found for both premenopausal and postmenopausal females, and this relationship remained significant after covariate adjustment in the group of postmenopausal females (supplementary table 24).

## Discussion

Our study has several novel findings. First, we found a significant sex interaction in the relationship between shift work frequency and prevalent moderate–severe asthma. Second, we showed that females, and not males, are at higher risk of moderate–severe asthma if they are night shift workers, compared with dayworkers, and this risk also increases with both number of monthly night shifts and longer lifetime duration of night shift work. Third, we identified a significant sex interaction in relationships between night shift work frequency and “any asthma” and “experiencing wheeze or whistling in the chest in the last year”. Fourth, we showed data suggesting that HRT may offer some protection from moderate–severe asthma in postmenopausal female permanent night shift workers. Our findings have important implications for female shift workers and should be considered in asthma clinics and in public health guidance.

To date, to our knowledge, no previous study has investigated sex differences in the relationship between shift work and asthma.

An important potential mechanism that could explain the sex–shift work interaction is differences in sex hormone levels. We demonstrated that higher levels of testosterone and SHBG appeared to be protective against asthma in both males and females, as shown previously [27]. We showed no significant relationships between oestradiol levels and prevalent moderate–severe asthma. Adding testosterone, SHBG and oestradiol as covariates in our models had no clear impact on the sex–shift work interaction for the relationship with asthma, suggesting that sex hormones might have limited roles in explaining the observed sex differences. However, there are some important caveats to consider in relation to our findings: 1) there were no time-of-sampling data for testosterone, 2) there were no data on time of menstrual cycle for oestradiol and 3) 80% of oestradiol levels were below the detectable level, possibly due to the age range of females studied. Such missing data reduced our statistical power to explore whether sex hormones modified the risk of shift work-related asthma in females.

We found that female participants who had had a hysterectomy±oophorectomy had a higher likelihood of moderate–severe asthma than premenopausal females. This has been reported previously [28], and it may be that these surgical procedures are acting as a marker for shorter lifetime exposure to oestrogen [28].

We showed that postmenopausal and premenopausal permanent night shift workers had higher odds of moderate–severe asthma than corresponding dayworkers, but only premenopausal females had a significant interaction between sex and shift work frequency; these findings attenuated after covariate adjustment and were similar after the addition of females over the age of 50 into the postmenopausal group (supplementary tables 20 and 21). However, when postmenopausal females taking HRT or OCP were excluded, female postmenopausal permanent night shift workers had 1.9-fold higher odds of moderate–severe asthma than female dayworkers, and a significant sex interaction was observed across all models (supplementary table 22). Taken together, this suggests that HRT or OCP use in postmenopausal females could be protective for moderate–severe asthma in the context of shift work.

Previous studies investigating the effect of menopause on asthma have come to different conclusions: some suggest that the menopause is protective for asthma [29, 30] and others show the opposite [31]. The role of HRT in asthma is also currently unclear; several observational studies, including a prospective study with UK Biobank participants [28], have suggested that HRT might have adverse effects on asthma risk [28–30, 32]. However, the largest prospective study performed to date showed that previous or current HRT use was associated with a 17–21% lower risk of developing asthma, and that longer use of HRT was associated with a lower risk of incident asthma in a dose-dependent manner [33]. Other recent data suggest that the type of HRT used (systemic *versus* local, oestrogen *versus* progesterone) might be important in determining whether HRT is a risk for, or protective against, asthma [34].

Our results would suggest that, in postmenopausal shift workers, HRT might be protective for night shift work-related asthma; however, further research is needed to test this hypothesis in prospective studies and randomised controlled trials (RCTs). These studies could test whether HRT protects against the development of asthma or reduces the severity of asthma in both dayworkers and in shift workers. It is currently unclear if shift work-related asthma risk would respond differently to HRT than asthma risk in dayworkers.

Previous studies have shown that the highest risk of HRT is in people using conjugated oestrogens [29, 35], who have a low BMI [32, 36] and are non-smokers [35]. Our results also suggest that BMI and smoking moderate the relationship between sex and asthma. We cannot comment on the type of HRT used by females in the UK Biobank.

We have shown previously that having an extreme chronotype (*i.e.* a definite preference for mornings or evenings) is associated with prevalent asthma [15]. We know that males and females have differing distributions of chronotype, with women tending to have earlier chronotypes; therefore, this provides a potential mechanism for the sex differences we observed. However, we found higher likelihood of moderate–severe asthma in extreme chronotypes, in both males and females, and no evidence of a sex–chronotype interaction on risk for asthma. Including chronotype in models linking shift work to moderate–severe asthma made no difference to results in either males or females. Therefore, we conclude that chronotype does not appear to explain the sex differences observed in the shift work–asthma relationships.

Attenuation in our models (particularly between models 2 and 3) suggests some effect of potential mediator variables on the sex differences observed. These variables, smoking, sleep duration and BMI, differed between the sexes (tables 1 and 2), with females having a higher increase of average BMI and proportion of current smokers between shift work groups and dayworkers than those observed in males. However, these moderators did not account for all of the effect observed, as sex–shift work interactions remained significant in model 3 (supplementary table 2).

Our study has several strengths. First, this is the first study to evaluate sex differences in the relationship between shift work frequency and asthma, an important topic from a public health perspective. Second, we did this in a large cohort (n=502 540) of individuals with linked health and socioeconomic variables. Third, we employed a robust definition of moderate–severe asthma [15], likely to capture those with active disease. Fourth, we employed a staged modelling strategy accounting for important confounders. We included potential mediating variables in our final models to provide some indication of their role in the observed relationships. These models accounted for the effects of risk factors that 1) showed sex differences, 2) were linked to shift work and 3) could have a causal role in asthma (sleep duration, smoking and BMI; tables 1 and 2). Finally, we explored potential mechanisms explaining how our results linked to differing chronotypes and sex hormone levels.

Our study has some limitations. First, residual confounding could affect our results. For example, undefined occupational exposures could have effects on prevalent asthma, especially when considering the different types of work undertaken by males and females (e.g. males doing more physical work and females more personal care and service work; see supplementary table 25). However, we took steps to mitigate for this effect by including, in models 2 and 3, covariates that indicated jobs with high asthma risk or where a medical assessment for asthma was undertaken prior to employment. Second, in some subgroups of participants, sample sizes were small, resulting in limited power. Third, causal inference in a cross-sectional study is not possible. Fourth, we potentially underestimate the detrimental effects of shift work on asthma due to the “healthy worker effect”: people leaving employment after becoming unwell as a result of their shift work and therefore not being present in the data. Lastly, the generalisability of our results may be limited because UK Biobank participants are generally healthier than the background population and have limited age, ethnic and social diversity [15].

Despite these limitations, our findings could have significant public health and clinical implications. The ability to reduce the risk of females developing asthma through modification of work schedules or through the introduction of HRT at menopause could have significant health and economic benefits.

### Conclusion

Our study shows that the higher likelihood of moderate–severe asthma among shift workers, compared with dayworkers, is present for females but not for males. Future large RCTs are needed to investigate whether HRT might be protective in postmenopausal female shift workers, who are an increasingly large proportion of our workforce.

## Supplementary material

**Please note:** supplementary material is not edited by the Editorial Office, and is uploaded as it has been supplied by the author. Supplementary material 00137-2025.SUPPLEMENT
