# Supplementary material for: Increased risk of asthma in female night shift workers
Source: ERJ Open Res. 2025 Nov 17;11(6):00137-2025. doi: 10.1183/23120541.00137-2025 (PMC12621128; doi:10.1183/23120541.00137-2025)
Supplement: Supplementary file 1 [file 00137-2025.SUPPLEMENT.pdf]

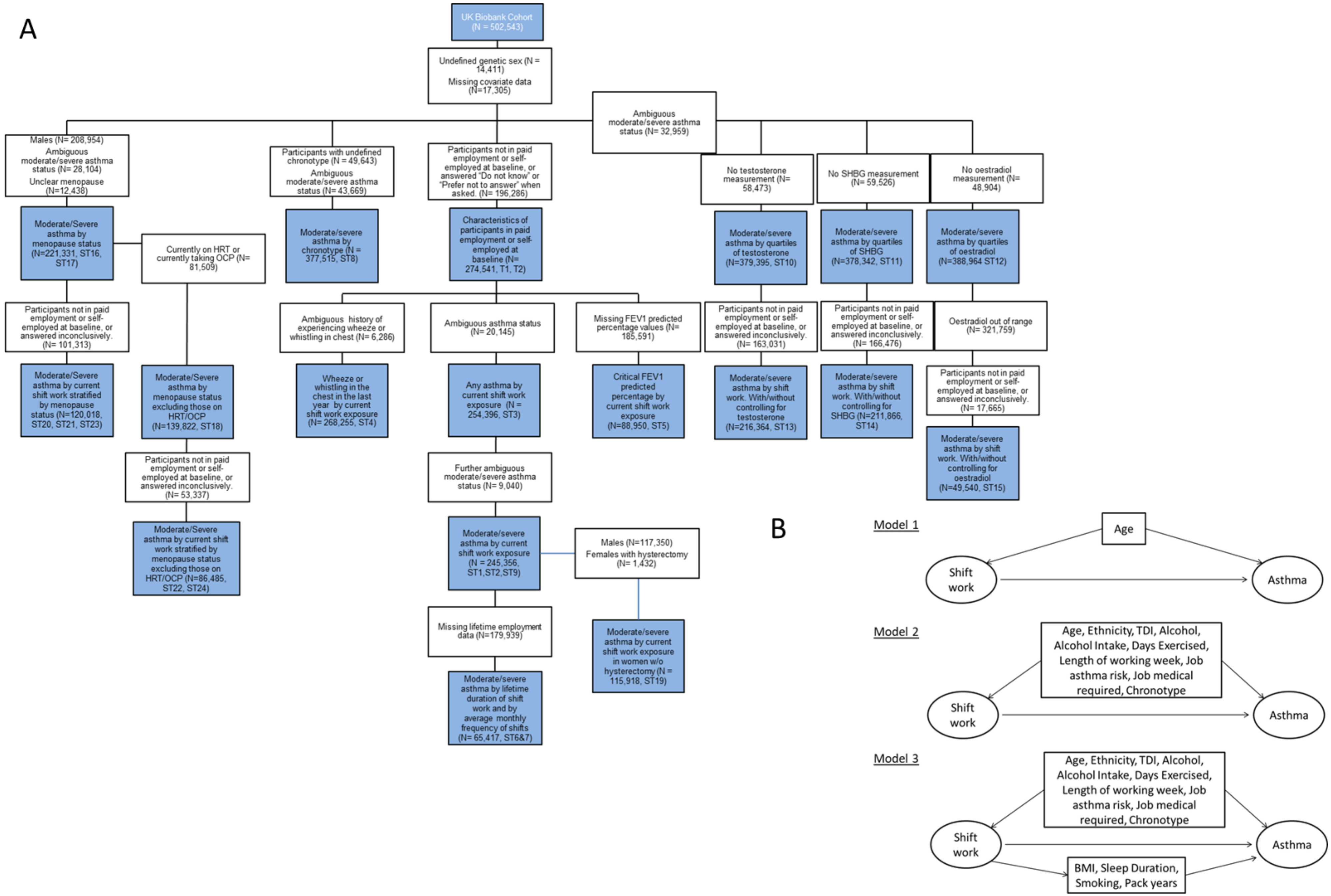

Supplementary Figure 1: A. STROBE diagram of data used in analyses B. Directed acrylic graphs of models used.

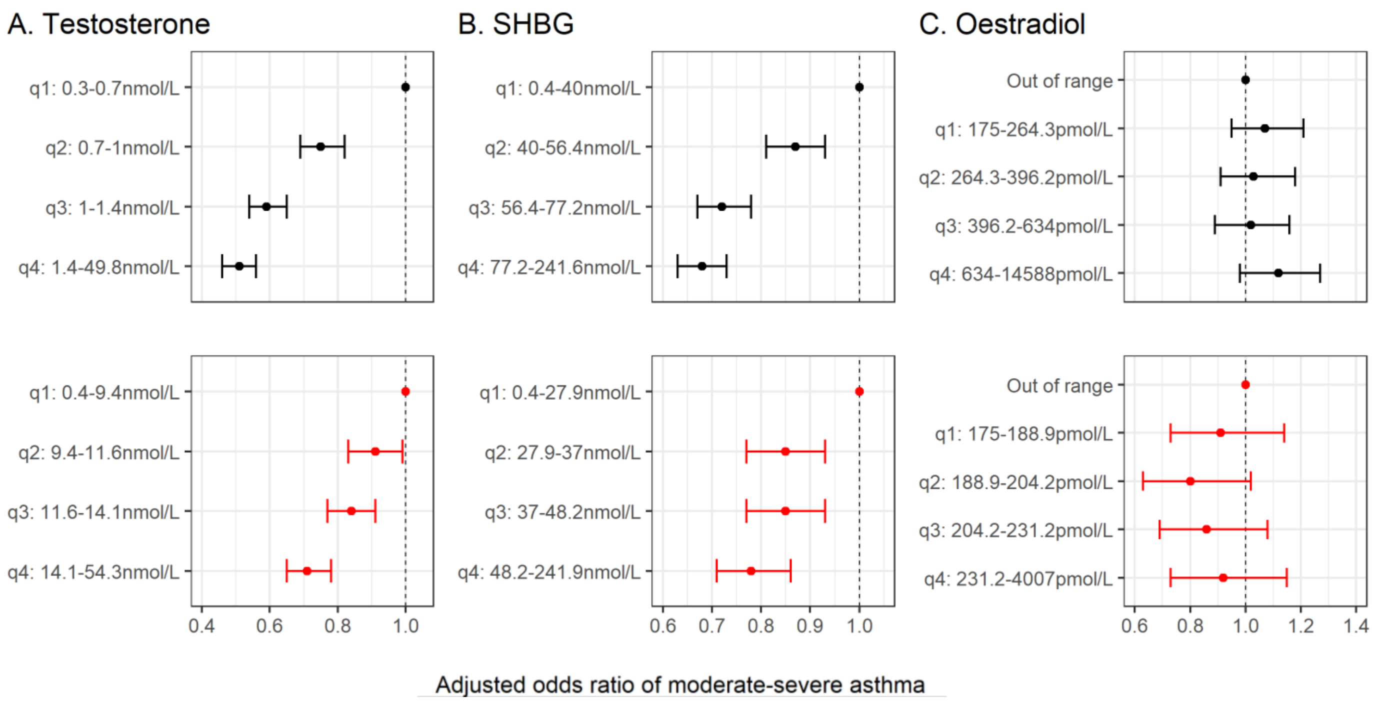

Supplementary figure 2. Effect of sex hormones on moderate-severe asthma, model 2. Female (top), male (bottom). Adjusted for covariates in Model 2: age, ethnicity, Townsend deprivation index, alcohol status, daily alcohol intake, days exercised (walked, moderate and vigorous), length of working week, job asthma risk, job medical required and chronotype.

Supplementary table 1: Mediated effect (95% CI) on moderate-severe/shift work relationship in females when potential mediators are added (N = 245,356)

Mediator	Mediated effect (95% CI)	Percentage of total effect mediated	p-value
BMI	0.0020 (0.0015-0.0025)	21.5%	<0.001
Pack years	0.0008 (0.0006-0.0012)	8.7%	<0.001
Sleep Duration	0.0005 (0.0003-0.0008)	5.4%	<0.001
Sleep Medication	0.0004 (0.0002-0.0007)	4.4%	<0.001
Smoking status	0.0003 (0.0001-0.0005)	3.2%	<0.001

Model 2 covariates: age, alcohol status, daily alcohol intake, ethnicity, Townsend deprivation index, days exercised (walked, moderate and vigorous), chronotype, length of working week, job asthma risk and job medical required.

Supplementary table 2: Adjusted odds (95% CI) of moderate-severe asthma by current shift work exposure with day workers as referent, stratified by sex. (n=245,356)

Female/Male	Current work schedule				Sex-shift work interaction
	Day workers	Shift work, but never or rarely night shifts	Irregular shift work including nights	Permanent night shift work	
Total cases (% of total sample size)	2,254 (2.07%)<br>1,469 (1.56%)	262 (2.39%)<br>161 (1.62%)	139 (2.38%)<br>139 (1.43%)	73 (3.13%)<br>52 (1.38%)	
Total sample size	108,849<br>93,937	10,983<br>9,941	5,841<br>9,709	2,333<br>3,763	
Model 1: Age-adjusted	Female referent<br>Male referent	**1.16 (1.02-1.32)**<br>1.05 (0.89-1.23)	1.18 (0.99-1.40)<br>0.94 (0.79-1.13)	**1.54 (1.22-1.95)**<br>0.91 (0.69-1.20)	**0.01**
Model 2: Multivariable-adjusted	Female referent<br>Male referent	1.13 (0.99-1.28)<br>1.08 (0.92-1.28)	1.19 (0.99-1.41)<br>0.98 (0.82-1.17)	**1.50 (1.18-1.91)**<br>0.95 (0.72-1.26)	**0.01**
Model 3: Model 2 covariates + potential moderators	Female referent<br>Male referent	1.05 (0.92-1.20)<br>1.05 (0.89-1.24)	1.10 (0.92-1.31)<br>0.95 (0.80-1.14)	**1.30 (1.02-1.66)**<br>0.90 (0.68-1.20)	**0.01**

Model 2 covariates: age, ethnicity, Townsend deprivation index, alcohol status, daily alcohol intake, days exercised (walked, moderate and vigorous), length of working week, chronotype, job asthma risk and job medical required. Model 3 data are adjusted for Model 2 covariates plus potential moderators sleep duration, sleep medication, smoking status, smoking pack years and BMI.

Supplementary table 3: Adjusted odds (95% CI) of any asthma by current shift work exposure with day workers as referent, stratified by sex. (n=254,396)

Female/Male	Current work schedule				Sex-shift work interaction
	Day workers	Shift work, but never or rarely night shifts	Irregular shift work including nights	Permanent night shift work	
Total cases (% of total sample size)	6,458 (5.71%)<br>4,715 (4.85%)	730 (6.37%)<br>508 (4.94%)	380 (6.25%)<br>450 (4.49%)	163 (6.73%)<br>185 (4.75%)	
Total sample size	113,053<br>97,183	11,451<br>10,288	6,082<br>10,020	2,423<br>3,896	
Model 1: Age-adjusted	Female referent<br>Male referent	**1.12 (1.04-1.22)**<br>1.01 (0.92-1.11)	1.09 (0.98-1.22)<br>**0.89 (0.81-0.99)**	**1.19 (1.01-1.40)**<br>0.95 (0.81-1.10)	**0.01**
Model 2: Multivariable-adjusted	Female referent<br>Male referent	**1.11 (1.02-1.20)**<br>1.05 (0.95-1.15)	1.10 (0.99-1.22)<br>0.94 (0.85-1.04)	1.17 (0.99-1.37)<br>1.00 (0.86-1.17)	**0.01**
Model 3: Model 2 covariates + potential moderators	Female referent<br>Male referent	1.06 (0.98-1.15)<br>1.04 (0.94-1.14)	1.04 (0.93-1.16)<br>0.94 (0.85-1.04)	1.06 (0.90-1.25)<br>0.99 (0.85-1.16)	**0.01**

Model 2 covariates: age, ethnicity, Townsend deprivation index, alcohol status, daily alcohol intake, days exercised (walked, moderate and vigorous), length of working week, chronotype, job asthma risk and job medical required. Model 3 data are adjusted for Model 2 covariates plus potential moderators sleep duration, sleep medication, smoking status, smoking pack years and BMI.

Supplementary table 4: Adjusted odds (95% CI) of experiencing wheeze or whistling in the chest within the last year by current shift work exposure with day workers as referent, stratified by sex. (n=268,255)

Female/Male	Current work schedule				Sex-shift work interaction
	Day workers	Shift work, but never or rarely night shifts	Irregular shift work including nights	Permanent night shift work	
Total cases (% of total sample size)	20,666 (17.25%)<br>19,936 (19.47%)	2,599 (21.50%)<br>2,503 (23.53%)	1,449 (22.55%)<br>2,501 (24.23%)	643 (25.10%)<br>993 (24.83%)	
Total sample size	119,807<br>102,414	12,087<br>10,639	6,425<br>10,322	2,562<br>3,999	
Model 1: Age-adjusted	Female referent<br>Male referent	**1.31 (1.26-1.38)**<br>**1.27 (1.21-1.33)**	**1.41 (1.33-1.50)**<br>**1.31 (1.25-1.38)**	**1.61 (1.47-1.77)**<br>**1.36 (1.26-1.46)**	**0.03**
Model 2: Multivariable-adjusted	Female referent<br>Male referent	**1.26 (1.21-1.32)**<br>**1.24 (1.18-1.30)**	**1.36 (1.28-1.44)**<br>**1.28 (1.22-1.35)**	**1.49 (1.36-1.64)**<br>**1.31 (1.22-1.41)**	**0.01**
Model 3: Model 2 covariates + potential moderators	Female referent<br>Male referent	**1.13 (1.08-1.19)**<br>**1.12 (1.06-1.17)**	**1.16 (1.09-1.24)**<br>**1.09 (1.03-1.14)**	**1.17 (1.06-1.29)**<br>**1.09 (1.01-1.18)**	0.11

Model 2 covariates: age, ethnicity, Townsend deprivation index, alcohol status, daily alcohol intake, days exercised (walked, moderate and vigorous), length of working week, chronotype, job asthma risk and job medical required. Model 3 data are adjusted for Model 2 covariates plus potential moderators sleep duration, sleep medication, smoking status, smoking pack years and BMI.

Supplementary table 5: Adjusted odds (95% CI) of having a critical (<80%) FEV1 predicted percentage by current shift work exposure with day workers as referent, stratified by sex. (n=88,950)

Female/Male	Current work schedule				Sex-shift work interaction
	Day workers	Shift work, but never or rarely night shifts	Irregular shift work including nights	Permanent night shift work	
Total cases (% of total sample size)	4,800 (11.67%)<br>4,368 (14.05%)	615 (15.04%)<br>547 (16.93%)	303 (13.89%)<br>529 (15.83%)	151 (17.00%)<br>237 (16.98%)	
Total sample size	41,140<br>31,094	4,088<br>3,230	2,181<br>3,341	888<br>1,396	
Model 1: Age-adjusted	Female referent<br>Male referent	**1.34 (1.23-1.47)**<br>**1.29 (1.17-1.42)**	**1.31 (1.15-1.48)**<br>**1.26 (1.14-1.39)**	**1.60 (1.34-1.91)**<br>**1.36 (1.18-1.57)**	0.52
Model 2: Multivariable-adjusted	Female referent<br>Male referent	**1.29 (1.17-1.41)**<br>**1.24 (1.13-1.37)**	**1.27 (1.12-1.45)**<br>**1.23 (1.11-1.36)**	**1.50 (1.25-1.80)**<br>**1.31 (1.14-1.52)**	0.61
Model 3: Model 2 covariates + potential moderators	Female referent<br>Male referent	**1.15 (1.05-1.27)**<br>**1.11 (1.01-1.23)**	1.08 (0.95-1.24)<br>1.04 (0.93-1.15)	1.16 (0.96-1.40)<br>1.12 (0.96-1.30)	0.86

Model 2 covariates: age, ethnicity, Townsend deprivation index, alcohol status, daily alcohol intake, days exercised (walked, moderate and vigorous), length of working week, chronotype, job asthma risk and job medical required. Model 3 data are adjusted for Model 2 covariates plus potential moderators sleep duration, sleep medication, smoking status, smoking pack years and BMI.

Supplementary table 6: Adjusted odds (95% CI) of moderate-severe asthma by average monthly number of night shifts, with none as referent, stratified by sex. (n=65,417)

Female/Male	Average monthly number of night shifts				p-value for trend
	None	< 5/month	5-10/month	≥ 10/month	
Total cases (% of total sample size)	545 (1.87%)<br>281 (1.37%)	44 (1.89%)<br>38 (1.70%)	60 (2.09%)<br>54 (1.58%)	53 (2.74%)<br>48 (1.57%)	
Total sample size	29,096<br>20,465	2,330<br>2,239	2,874<br>3,418	1,937<br>3,058	
Model 1: Age-adjusted	Female referent<br>Male referent	1.01 (0.74-1.38)<br>1.24 (0.88-1.75)	1.12 (0.85-1.46)<br>1.17 (0.87-1.57)	**1.47 (1.11-1.96)**<br>1.15 (0.84-1.56)	0.14<br>0.83
Model 2: Multivariable-adjusted	Female referent<br>Male referent	1.02 (0.74-1.39)<br>1.31 (0.92-1.85)	1.13 (0.86-1.48)<br>1.26 (0.93-1.72)	**1.44 (1.08-1.92)**<br>1.22 (0.88-1.68)	0.14<br>0.84
Model 3: Model 2 covariates + potential moderators	Female referent<br>Male referent	0.97 (0.71-1.33)<br>1.27 (0.90-1.81)	1.08 (0.82-1.42)<br>1.19 (0.87-1.63)	1.33 (1.00-1.78)<br>1.17 (0.85-1.62)	0.14<br>0.84

Model 2 covariates: age, ethnicity, Townsend deprivation index, alcohol status, daily alcohol intake, days exercised (walked, moderate and vigorous), length of working week, chronotype, job asthma risk and job medical required. Model 3 data are adjusted for Model 2 covariates plus potential moderators sleep duration, sleep medication, smoking status, smoking pack years and BMI.

Supplementary table 7: Adjusted odds (95% CI) of moderate-severe asthma by lifetime duration of shift work including nights, with none as referent, stratified by sex. (n=65,417)

Female/Male	Lifetime duration of shift work including nights				p-value for trend
	None	< 5 years	5-10 years	≥ 10 years	
Total cases (% of total sample size)	545 (1.87%)<br>283 (1.38%)	25 (1.86%)<br>24 (1.94%)	34 (2.18%)<br>29 (1.98%)	98 (2.36%)<br>85 (1.43%)	
Total sample size	29,180<br>20,551	1,347<br>1,239	1,557<br>1,461	4,153<br>5,929	
Model 1: Age-adjusted	Female referent<br>Male referent	0.99 (0.66-1.49)<br>1.42 (0.93-2.17)	1.17 (0.83-1.67)<br>1.46 (0.99-2.15)	**1.27 (1.02-1.58)**<br>1.05 (0.82-1.33)	**0.02**<br>0.83
Model 2: Multivariable-adjusted	Female referent<br>Male referent	0.97 (0.64-1.46)<br>1.48 (0.97-2.27)	1.19 (0.83-1.69)<br>**1.54 (1.04-2.28)**	**1.28 (1.03-1.59)**<br>1.11 (0.86-1.45)	**0.02**<br>0.82
Model 3: Model 2 covariates + potential moderators	Female referent<br>Male referent	0.93 (0.62-1.40)<br>1.47 (0.96-2.26)	1.13 (0.80-1.61)<br>**1.52 (1.02-2.25)**	1.20 (0.97-1.50)<br>1.05 (0.81-1.37)	**0.02**<br>0.83

Model 2 covariates: age, ethnicity, Townsend deprivation index, alcohol status, daily alcohol intake, days exercised (walked, moderate and vigorous), length of working week, chronotype, job asthma risk and job medical required. Model 3 data are adjusted for Model 2 covariates plus potential moderators sleep duration, sleep medication, smoking status, smoking pack years and BMI.

Supplementary table 8: Adjusted odds (95% CI) of moderate-severe asthma by chronotype with intermediate chronotype as referent, stratified by sex. (n=377,515)

Female/Male	Chronotype			Sex-chronotype interaction
	Intermediate	Definitely a morning person	Definitely an evening person	
Total cases (% of total sample size)	3,431 (2.53%)<br>2,101 (1.98%)	1,665 (2.86%)<br>974 (2.20%)	565 (3.18%)<br>373 (2.46%)	
Total sample size	135,840<br>106,135	58,290<br>44,312	17,756<br>15,182	
Model 1: Age-adjusted	Female referent<br>Male referent	**1.11 (1.05-1.18)**<br>**1.09 (1.01-1.17)**	**1.29 (1.18-1.41)**<br>**1.31 (1.17-1.47)**	0.96
Model 2: Multivariable-adjusted	Female referent<br>Male referent	**1.10 (1.03-1.17)**<br>**1.10 (1.02-1.19)**	**1.22 (1.11-1.34)**<br>**1.20 (1.08-1.35)**	0.91
Model 3: Model 2 covariates + potential moderators	Female referent<br>Male referent	**1.10 (1.04-1.17)**<br>**1.09 (1.01-1.18)**	1.08 (0.99-1.19)<br>**1.14 (1.02-1.28)**	0.75

Model 2 covariates: age, ethnicity, Townsend deprivation index, alcohol status, daily alcohol intake, days exercised (walked, moderate and vigorous), length of working week, job asthma risk and job medical required. Model 3 data are adjusted for Model 2 covariates plus potential moderators sleep duration, sleep medication, smoking status, smoking pack years and BMI.

Supplementary table 9: Adjusted odds (95% CI) of moderate-severe asthma by current shift work exposure with day workers as referent, stratified by sex. (n=245,356). Comparison of models with/without adjusting for chronotype.

Female/Male	Current work schedule				
	Day workers	Shift work, but never or rarely night shifts	Irregular shift work including nights	Permanent night shift work	Sex-shift work interaction
Total cases (% of total sample size)	2,254 (2.07%)<br>1,469 (1.56%)	262 (2.39%)<br>161 (1.62%)	139 (2.38%)<br>139 (1.43%)	73 (3.13%)<br>52 (1.38%)	
Total sample size	108,849<br>93,937	10,983<br>9,941	5,841<br>9,709	2,333<br>3,763	
Model 1: Age-adjusted	Female referent<br>Male referent	**1.16 (1.02-1.32)**<br>1.05 (0.89-1.23)	1.18 (0.99-1.40)<br>0.94 (0.79-1.13)	**1.54 (1.22-1.95)**<br>0.91 (0.69-1.20)	**0.01**
Model 1 + chronotype	Female referent<br>Male referent	**1.16 (1.02-1.32)**<br>1.05 (0.89-1.24)	1.18 (0.99-1.40)<br>0.94 (0.79-1.13)	**1.53 (1.21-1.94)**<br>0.91 (0.69-1.20)	**0.01**
Model 2 - chronotype	Female referent<br>Male referent	1.13 (0.99-1.28)<br>1.08 (0.92-1.28)	1.19 (1.00-1.42)<br>0.98 (0.82-1.17)	**1.51 (1.19-1.92)**<br>0.95 (0.72-1.26)	**0.01**
Model 2: Multivariable-adjusted	Female referent<br>Male referent	1.13 (0.99-1.28)<br>1.08 (0.92-1.28)	1.19 (0.99-1.41)<br>0.98 (0.82-1.17)	**1.50 (1.18-1.91)**<br>0.95 (0.72-1.26)	**0.01**
Model 3 - chronotype	Female referent<br>Male referent	1.05 (0.92-1.20)<br>1.05 (0.89-1.24)	1.10 (0.92-1.31)<br>0.95 (0.80-1.14)	**1.31 (1.03-1.67)**<br>0.91 (0.68-1.20)	**0.01**
Model 3: Model 2 covariates + potential moderators	Female referent<br>Male referent	1.05 (0.92-1.20)<br>1.05 (0.89-1.24)	1.10 (0.92-1.31)<br>0.95 (0.80-1.14)	**1.30 (1.02-1.66)**<br>0.90 (0.68-1.20)	**0.01**

Model 2 covariates: age, ethnicity, Townsend deprivation index, alcohol status, daily alcohol intake, days exercised (walked, moderate and vigorous), length of working week, chronotype, job asthma risk and job medical required. Model 3 data are adjusted for Model 2 covariates plus potential moderators sleep duration, sleep medication, smoking status, smoking pack years and BMI.

Supplementary table 10: Adjusted odds (95% CI) of moderate-severe asthma by testosterone with first quartile of testosterone as referent, stratified by sex. (n=379,395)

Female (n=189,029)	Testosterone quartile				p-value for trend
	q1: 0.4-0.7nmol/L	q2: 0.7-1.0nmol/L	q3: 1.0-1.4nmol/L	q4: 1.4-49.8nmol/L	
Total cases (% of total sample size)	1,309 (2.80%)	991 (2.10%)	797 (1.68%)	701 (1.47%)	
Total sample size	46,707	47,191	47,386	47,745	
Model 1: Age-adjusted OR (95% CI)	Female referent	**0.75 (0.69-0.81)**	**0.60 (0.55-0.66)**	**0.52 (0.48-0.58)**	**<0.01**
Model 2: Multivariable adjusted OR (95% CI)	Female referent	**0.75 (0.69-0.82)**	**0.59 (0.54-0.65)**	**0.51 (0.46-0.56)**	**<0.01**
Model 3: Model 2 covariates + potential moderators (95% CI)	Female referent	**0.73 (0.67-0.80)**	**0.57 (0.52-0.62)**	**0.46 (0.42-0.50)**	**<0.01**
Male (n=190,366)	q1: 0.4-9.4nmol/L	q2: 9.4-11.6nmol/L	q3: 11.6-14.1nmol/L	q4: 14.1-54.3nmol/L	
Total cases (% of total sample size)	1,185 (2.51%)	1,029 (2.16%)	937 (1.96%)	800 (1.67%)	
Total sample size	47,181	47,586	47,746	47,853	
Model 1: Age-adjusted OR (95% CI)	Male referent	**0.87 (0.80-0.95)**	**0.79 (0.73-0.87)**	**0.68 (0.62-0.75)**	**<0.01**
Model 2: Multivariable adjusted OR (95% CI)	Male referent	**0.91 (0.83-0.99)**	**0.84 (0.77-0.91)**	**0.71 (0.65-0.78)**	**<0.01**
Model 3: Model 2 covariates + potential moderators (95% CI)	Male referent	0.98 (0.89-1.07)	0.94 (0.86-1.04)	**0.82 (0.74-0.90)**	**<0.01**

Model 2 covariates: age, ethnicity, Townsend deprivation index, alcohol status, daily alcohol intake, days exercised (walked, moderate and vigorous), length of working week, chronotype, job asthma risk and job medical required. Model 3 data are adjusted for Model 2 covariates plus potential moderators sleep duration, sleep medication, smoking status, smoking pack years and BMI.

Supplementary table 11: Adjusted odds (95% CI) of moderate-severe asthma by SHBG with first quartile of SHBG as referent, stratified by sex. (n=378,342)

Female (n=202,186)	SHBG quartile				p-value for trend
	q1: 0.4-40.0nmol/L	q2: 40.0-56.4nmol/L	q3: 56.4-77.2nmol/L	q4: 77.2-241.6nmol/L	
Total cases (% of total sample size)	1,718 (3.45%)	1,441 (2.85%)	1,177 (2.31%)	1,096 (2.15%)	
Total sample size	49,726	50,502	51,011	50,947	
Model 1: Age-adjusted OR (95% CI)	Female referent	**0.82 (0.76-0.88)**	**0.67 (0.62-0.72)**	**0.63 (0.58-0.68)**	**<0.01**
Model 2: Multivariable adjusted OR (95% CI)	Female referent	**0.87 (0.81-0.93)**	**0.72 (0.67-0.78)**	**0.68 (0.63-0.73)**	**<0.01**
Model 3: Model 2 covariates + potential moderators (95% CI)	Female referent	1.02 (0.95-1.10)	0.94 (0.86-1.02)	0.96 (0.88-1.05)	**<0.01**
Male (n=176,156)	q1: 0.4-27.9nmol/L	q2: 27.9-37.0nmol/L	q3: 37.0-48.2nmol/L	q4: 48.2-241.9nmol/L	
Total cases (% of total sample size)	964 (2.21%)	876 (1.99%)	917 (2.08%)	912 (2.05%)	
Total sample size	43,555	44,001	44,141	44,459	
Model 1: Age-adjusted OR (95% CI)	Male referent	**0.83 (0.76-0.91)**	**0.83 (0.75-0.91)**	**0.78 (0.70-0.85)**	0.68
Model 2: Multivariable adjusted OR (95% CI)	Male referent	**0.85 (0.77-0.93)**	**0.85 (0.77-0.93)**	**0.78 (0.71-0.86)**	0.81
Model 3: Model 2 covariates + potential moderators (95% CI)	Male referent	0.91 (0.83-1.01)	0.92 (0.83-1.02)	**0.90 (0.81-0.99)**	0.58

Model 2 covariates: age, ethnicity, Townsend deprivation index, alcohol status, daily alcohol intake, days exercised (walked, moderate and vigorous), length of working week, chronotype, job asthma risk and job medical required. Model 3 data are adjusted for Model 2 covariates plus potential moderators sleep duration, sleep medication, smoking status, smoking pack years and BMI.

Supplementary table 12: Adjusted odds (95% CI) of moderate-severe asthma by oestradiol with participants recording measurements below the reportable range (<175 pmol/L) as referent, stratified by sex. (n=388,964)

Female (n=209,700)	Oestradiol quartile					p-value for trend
	Out of range	q1: 175.0-264.3pmol/L	q2: 264.3-396.2pmol/L	q3: 396.2-634.0pmol/L	q4: 634.0-14,588pmol/L	
Total cases (% of total sample size)	4,410 (2.77%)	316 (2.50%)	286 (2.26%)	272 (2.15%)	298 (2.35%)	
Total sample size	159,076	12,630	12,667	12,634	12,693	
Model 1: Age- adjusted OR (95% CI)	Female referent	1.09 (0.97-1.23)	1.03 (0.91-1.18)	1.01 (0.88-1.15)	1.11 (0.97-1.26)	**<0.01**
Model 2: Multivariable adjusted OR (95% CI)	Female referent	1.07 (0.95-1.21)	1.03 (0.91-1.18)	1.02 (0.89-1.16)	1.12 (0.98-1.27)	**<0.01**
Model 3: Model 2 covariates + potential moderators (95% CI)	Female referent	1.06 (0.93-1.19)	1.04 (0.92-1.19)	1.05 (0.91-1.20)	**1.20 (1.05-1.37)**	**<0.01**
Male (n=179,264)	Out of range	q1: 175.0-188.9pmol/L	q2: 188.9-204.2pmol/L	q3: 204.2-231.2pmol/L	q4: 231.2-4,007pmol/L	
Total cases (% of total sample size)	3,426 (2.11%)	82 (1.97%)	71 (1.71%)	78 (1.87%)	82 (2.00%)	
Total sample size	162,683	4,161	4,140	4,173	4,107	
Model 1: Age-adjusted OR (95% CI)	Male referent	0.93 (0.75-1.16)	0.80 (0.63-1.02)	0.88 (0.70-1.11)	0.97 (0.77-1.21)	0.30
Model 2: Multivariable adjusted OR (95% CI)	Male referent	0.91 (0.73-1.14)	0.80 (0.63-1.02)	0.86 (0.69-1.08)	0.92 (0.73-1.15)	0.34
Model 3: Model 2 covariates + potential moderators (95% CI)	Male referent	0.94 (0.75-1.19)	0.81 (0.64-1.04)	0.83 (0.66-1.06)	0.92 (0.73-1.16)	0.35

p-value for trend includes participants out of range of oestradiol measurements by setting them to the minimum detectable level of 175 pmol/L. Model 2 covariates: age, ethnicity, Townsend deprivation index, alcohol status, daily alcohol intake, days exercised (walked, moderate and vigorous), length of working week, chronotype, job asthma risk and job medical required. Model 3 data are adjusted for Model 2 covariates plus potential moderators sleep duration, sleep medication, smoking status, smoking pack years and BMI.

Supplementary table 13: Adjusted odds (95% CI) of moderate-severe asthma by current shift work exposure, stratified by sex with day workers as referent. Comparison of models with/without adjusting for testosterone, in participants with measurements for testosterone. (n=216,364)

Female/Male	Current work schedule				
	Day workers	Shift work, but never or rarely night shifts	Irregular shift work including nights	Permanent night shift work	Sex-shift work interaction
Total cases (% of total sample size)	1,466 (1.64%)<br>1,381 (1.55%)	179 (1.98%)<br>152 (1.61%)	103 (2.12%)<br>129 (1.40%)	44 (2.32%)<br>51 (1.43%)	
Total sample size	89,575<br>88,848	9,018<br>9,412	4,854<br>9,190	1,900<br>3,567	
Model 1: Age-adjusted	Female referent<br>Male referent	**1.22 (1.04-1.42)**<br>1.05 (0.89-1.25)	**1.31 (1.07-1.60)**<br>0.93 (0.78-1.12)	**1.43 (1.05-1.93)**<br>0.95 (0.72-1.26)	**0.01**
Model 1 + testosterone	Female referent<br>Male referent	**1.21 (1.04-1.42)**<br>1.05 (0.89-1.25)	**1.30 (1.07-1.60)**<br>0.93 (0.78-1.12)	**1.43 (1.06-1.94)**<br>0.95 (0.72-1.26)	**0.01**
Model 2: Multivariable-adjusted	Female referent<br>Male referent	**1.20 (1.02-1.40)**<br>1.09 (0.92-1.29)	**1.32 (1.08-1.62)**<br>0.97 (0.81-1.17)	**1.41 (1.04-1.91)**<br>0.99 (0.74-1.32)	**<0.01**
Model 2 + testosterone	Female referent<br>Male referent	**1.20 (1.02-1.40)**<br>1.09 (0.92-1.29)	**1.32 (1.08-1.62)**<br>0.97 (0.80-1.16)	**1.42 (1.04-1.93)**<br>0.99 (0.75-1.32)	**<0.01**
Model 3: Model 2 covariates + potential moderators	Female referent<br>Male referent	1.10 (0.94-1.29)<br>1.06 (0.89-1.25)	1.21 (0.99-1.49)<br>0.94 (0.78-1.13)	1.19 (0.87-1.63)<br>0.94 (0.71-1.25)	**0.01**
Model 3 + testosterone	Female referent<br>Male referent	1.09 (0.93-1.28)<br>1.06 (0.89-1.26)	1.20 (0.98-1.48)<br>0.94 (0.78-1.13)	1.19 (0.87-1.62)<br>0.95 (0.71-1.26)	**0.01**

Model 2 covariates: age, ethnicity, Townsend deprivation index, alcohol status, daily alcohol intake, days exercised (walked, moderate and vigorous), length of working week, chronotype, job asthma risk and job medical required. Model 3 data are adjusted for Model 2 covariates plus potential moderators sleep duration, sleep medication, smoking status, smoking pack years and BMI.

Supplementary table 14: Adjusted odds (95% CI) of moderate-severe asthma by current shift work exposure, stratified by sex with day workers as referent. Comparison of models with/without adjusting for SHBG, in participants with measurements for SHBG. (n=211,866)

Female/Male	Current work schedule				Sex-shift work interaction
	Day workers	Shift work, but never or rarely night shifts	Irregular shift work including nights	Permanent night shift work	
Total cases (% of total sample size)	1,930 (2.08%)<br>1,279 (1.56%)	237 (2.51%)<br>143 (1.65%)	119 (2.40%)<br>120 (1.41%)	62 (3.10%)<br>48 (1.45%)	
Total sample size	92,864<br>82,070	9,446<br>8,691	4,956<br>8,532	1,999<br>3,308	
Model 1: Age-adjusted	Female referent<br>Male referent	**1.21 (1.06-1.39)**<br>1.07 (0.90-1.27)	1.19 (0.98-1.43)<br>0.93 (0.77-1.12)	**1.52 (1.18-1.97)**<br>0.96 (0.72-1.28)	**0.03**
Model 1 + SHBG	Female referent<br>Male referent	**1.20 (1.05-1.38)**<br>1.07 (0.90-1.27)	1.17 (0.97-1.42)<br>0.93 (0.77-1.12)	**1.51 (1.17-1.95)**<br>0.96 (0.72-1.28)	**0.03**
Model 2: Multivariable-adjusted	Female referent<br>Male referent	**1.18 (1.03-1.36)**<br>1.11 (0.93-1.32)	1.19 (0.99-1.44)<br>0.97 (0.80-1.17)	**1.50 (1.15-1.94)**<br>1.00 (0.75-1.34)	**0.02**
Model 2 + SHBG	Female referent<br>Male referent	**1.18 (1.02-1.35)**<br>1.11 (0.93-1.32)	1.18 (0.98-1.43)<br>0.97 (0.80-1.17)	**1.49 (1.15-1.93)**<br>1.00 (0.75-1.34)	**0.02**
Model 3: Model 2 covariates + potential moderators	Female referent<br>Male referent	1.10 (0.96-1.26)<br>1.08 (0.90-1.28)	1.11 (0.92-1.34)<br>0.94 (0.77-1.14)	**1.30 (1.00-1.69)**<br>0.96 (0.71-1.28)	**0.03**
Model 3 + SHBG	Female referent<br>Male referent	1.10 (0.96-1.26)<br>1.08 (0.90-1.28)	1.11 (0.92-1.34)<br>0.94 (0.77-1.14)	**1.30 (1.00-1.69)**<br>0.96 (0.71-1.28)	**0.03**

Model 2 covariates: age, ethnicity, Townsend deprivation index, alcohol status, daily alcohol intake, days exercised (walked, moderate and vigorous), length of working week, chronotype, job asthma risk and job medical required. Model 3 data are adjusted for Model 2 covariates plus potential moderators sleep duration, sleep medication, smoking status, smoking pack years and BMI.

Supplementary table 15: Adjusted odds (95% CI) of moderate-severe asthma by current shift work exposure, stratified by sex with day workers as referent. Comparison of models with/without adjusting for oestradiol, in participants with measurements for oestradiol. (n=49,540)

Female/Male	Current work schedule				Oestradiol-shift work interaction
	Day workers	Shift work, but never or rarely night shifts	Irregular shift work including nights	Permanent night shift work	
Total cases (% of total sample size)	652 (1.92%)<br>116 (1.57%)	97 (2.85%)<br>12 (1.38%)	54 (2.55%)<br>9 (1.11%)	18 (2.37%)<br>4 (1.27%)	
Total sample size	33,967<br>7,372	3,404<br>868	2,120<br>809	761<br>314	
Model 1: Age-adjusted	Female referent<br>Male referent	**1.50 (1.21-1.86)**<br>0.87 (0.48-1.58)	**1.36 (1.03-1.80)**<br>0.68 (0.34-1.35)	1.25 (0.78-2.02)<br>0.78 (0.29-2.13)	0.16
Model 1 + oestradiol	Female referent<br>Male referent	**1.50 (1.21-1.86)**<br>0.87 (0.48-1.58)	**1.36 (1.03-1.80)**<br>0.68 (0.34-1.36)	1.25 (0.78-2.02)<br>0.78 (0.28-2.13)	0.16
Model 2: Multivariable-adjusted	Female referent<br>Male referent	**1.47 (1.18-1.82)**<br>0.91 (0.50-1.67)	**1.37 (1.03-1.82)**<br>0.70 (0.35-1.40)	1.25 (0.78-2.02)<br>0.81 (0.29-2.23)	0.16
Model 2 + oestradiol	Female referent<br>Male referent	**1.47 (1.18-1.83)**<br>0.91 (0.50-1.67)	**1.37 (1.03-1.82)**<br>0.70 (0.35-1.41)	1.25 (0.78-2.02)<br>0.80 (0.29-2.22)	0.16
Model 3: Model 2 covariates + potential moderators	Female referent<br>Male referent	**1.34 (1.08-1.68)**<br>0.95 (0.51-1.74)	1.28 (0.96-1.71)<br>0.73 (0.37-1.48)	1.04 (0.64-1.71)<br>0.83 (0.30-2.29)	0.18
Model 3 + oestradiol	Female referent<br>Male referent	**1.35 (1.08-1.68)**<br>0.95 (0.52-1.74)	1.29 (0.97-1.71)<br>0.73 (0.36-1.47)	1.04 (0.63-1.70)<br>0.83 (0.30-2.28)	0.18

Model 2 covariates: age, ethnicity, Townsend deprivation index, alcohol status, daily alcohol intake, days exercised (walked, moderate and vigorous), length of working week, chronotype, job asthma risk and job medical required. Model 3 data are adjusted for Model 2 covariates plus potential moderators sleep duration, sleep medication, smoking status, smoking pack years and BMI.

Supplementary table 16: Adjusted odds (95% CI) of having moderate-severe asthma by self-reported menopause status with premenopausal females as referent. (n=221,331)

	Menopause Status					
	Pre-menopausal	Post-menopausal	Unsure – had hysterectomy	Unsure – had hysterectomy (inc. removal of ovaries)	Unsure – other reason	Prefer not to answer
Total cases (% of total sample size)	1,150 (2.04%)	3,939 (2.68%)	121 (3.61%)	135 (3.56%)	247 (2.43%)	16 (3.84%)
Total sample size	56,363	147,245	3,353	3,794	10,159	417
Model 1: Age-adjusted	Referent	1.03 (0.93-1.14)	**1.64 (1.35-1.99)**	**1.52 (1.26-1.84)**	1.09 (0.95-1.26)	**1.98 (1.19-3.31)**
Model 2: Multivariable-adjusted	Referent	1.06 (0.96-1.17)	**1.56 (1.28-1.90)**	**1.46 (1.20-1.76)**	1.12 (0.97-1.30)	1.53 (0.91-2.59)
Model 3: Model 2 covariates + potential moderators	Referent	0.99 (0.90-1.09)	**1.34 (1.10-1.63)**	**1.24 (1.02-1.50)**	1.02 (0.88 - 1.18)	1.52 (0.90-2.57)

Model 2 covariates: age, ethnicity, Townsend deprivation index, alcohol status, daily alcohol intake, days exercised (walked, moderate and vigorous), length of working week, chronotype, job asthma risk and job medical required. Model 3 data are adjusted for Model 2 covariates plus potential moderators sleep duration, sleep medication, smoking status, smoking pack years and BMI.

Supplementary table 17: Adjusted odds (95% CI) of having moderate-severe asthma by menopause status, defined as self-reported or older than 50, with premenopausal females as referent. (n=221,331)

	Menopause Status					
	Pre-menopausal	Post-menopausal	Unsure – had hysterectomy	Unsure – had hysterectomy (inc. removal of ovaries)	Unsure – other reason	Prefer not to answer
Total cases (% of total sample size)	956 (2.06%)	4,306 (2.64%)	121 (3.61%)	135 (3.56%)	88 (2.00%)	2 (1.38%)
Total sample size	46,302	163,341	3,353	3,794	4,396	145
Model 1: Age-adjusted	Referent	0.95 (0.85-1.06)	**1.54 (1.26-1.88)**	**1.42 (1.17-1.72)**	0.92 (0.73-1.16)	0.76 (0.19-3.06)
Model 2: Multivariable-adjusted	Referent	1.02 (0.91-1.13)	**1.51 (1.24-1.85)**	**1.40 (1.15-1.71)**	0.91 (0.73-1.15)	0.54 (0.13-2.24)
Model 3: Model 2 covariates + potential moderators	Referent	0.92 (0.83-1.03)	**1.26 (1.03-1.55)**	1.17 (0.96-1.42)	0.82 (0.65 - 1.04)	0.53 (0.13-2.22)

Model 2 covariates: age, ethnicity, Townsend deprivation index, alcohol status, daily alcohol intake, days exercised (walked, moderate and vigorous), length of working week, chronotype, job asthma risk and job medical required. Model 3 data are adjusted for Model 2 covariates plus potential moderators sleep duration, sleep medication, smoking status, smoking pack years and BMI.

Supplementary table 18: Adjusted odds (95% CI) of having moderate-severe asthma by menopause status, defined as self-reported or older than 50, with premenopausal females as referent. Participants on HRT or currently taking OCP are excluded. (n=139,822)

	Menopause Status					
	Pre-menopausal	Post-menopausal	Unsure – had hysterectomy	Unsure – had hysterectomy (inc. removal of ovaries)	Unsure – other reason	Prefer not to answer
Total cases (% of total sample size)	849 (2.02%)	1,929 (2.11%)	55 (2.62%)	26 (3.12%)	69 (1.95%)	1 (1.41%)
Total sample size	42,023	91,250	2,098	833	3,547	71
Model 1: Age-adjusted	Referent	0.88 (0.77-1.00)	1.23 (0.93-1.64)	1.36 (0.90-2.05)	0.92 (0.71-1.18)	0.78 (0.11-5.60)
Model 2: Multivariable-adjusted	Referent	0.94 (0.82-1.07)	1.22 (0.92-1.62)	1.33 (0.88-2.01)	0.91 (0.70-1.18)	0.55 (0.08-4.02)
Model 3: Model 2 covariates + potential moderators	Referent	0.89 (0.78-1.01)	1.03 (0.77-1.38)	1.05 (0.69-1.62)	0.81 (0.62 - 1.05)	0.60 (0.08-4.40)

Model 2 covariates: age, ethnicity, Townsend deprivation index, alcohol status, daily alcohol intake, days exercised (walked, moderate and vigorous), length of working week, chronotype, job asthma risk and job medical required. Model 3 data are adjusted for Model 2 covariates plus potential moderators sleep duration, sleep medication, smoking status, smoking pack years and BMI.

Supplementary table 19: Adjusted odds (95% CI) of moderate-severe asthma by current shift work exposure in females who have not had a hysterectomy with day workers as referent (n=115,918).

	Current work schedule			
	Day workers	Shift work, but never or rarely night shifts	Irregular shift work including nights	Permanent night shift work
Total cases (% of total sample size)	1,961 (1.98%)	226 (2.31%)	115 (2.19%)	60 (2.93%)
Total sample size	98,849	9,765	5,253	2,051
Model 1: Age-adjusted	Referent	**1.17 (1.02-1.35)**	1.13 (0.93-1.37)	**1.50 (1.16-1.95)**
Model 2: Multivariable-adjusted	Referent	1.14 (0.99-1.32)	1.14 (0.94-1.39)	**1.48 (1.14-1.93)**
Model 3: Model 2 covariates + potential moderators	Referent	1.07 (0.93-1.23)	1.06 (0.87-1.28)	1.28 (0.98-1.68)

Model 2 covariates: age, ethnicity, Townsend deprivation index, alcohol status, daily alcohol intake, days exercised (walked, moderate and vigorous), length of working week, chronotype, job asthma risk and job medical required. Model 3 data are adjusted for Model 2 covariates plus potential moderators sleep duration, sleep medication, smoking status, smoking pack years and BMI.

Supplementary table 20: Adjusted odds (95% CI) of having moderate-severe asthma by current shift work exposure with day workers as referent, stratified by self-reported menopause status. (n=120,018)

Premenopausal<br>Postmenopausal<br>Hysterectomy	Current work schedule				Menopause status-shift work interaction
	Day workers	Shift work, but never or rarely night shifts	Irregular shift work including nights	Permanent night shift work	
Total cases (% of total sample size)	715 (1.83%)<br>1,103 (2.08%)<br>291 (2.94%)	93 (2.46%)<br>119 (2.24%)<br>36 (3.00%)	55 (2.27%)<br>52 (2.12%)<br>24 (4.20%)	25 (3.05%)<br>32 (2.96%)<br>12 (4.41%)	
Total sample size	39,083<br>53,104<br>9,910	3,778<br>5,320<br>1,200	2,427<br>2,451<br>572	819<br>1,082<br>272	
Model 1: Age-adjusted	Premenopausal referent<br>Postmenopausal referent<br>Hysterectomy referent	**1.35 (1.09-1.69)**<br>1.08 (0.90-1.31)<br>1.02 (0.72-1.45)	1.25 (0.94-1.64)<br>1.06 (0.80-1.41)<br>1.45 (0.95-2.22)	**1.69 (1.13-2.54)**<br>**1.47 (1.03-2.10)**<br>1.53 (0.85-2.75)	0.46
Model 2: Multivariable-adjusted	Premenopausal referent<br>Postmenopausal referent<br>Hysterectomy referent	**1.32 (1.06-1.65)**<br>1.06 (0.87-1.28)<br>0.98 (0.69-1.40)	1.28 (0.97-1.70)<br>1.08 (0.81-1.44)<br>1.49 (0.97-2.30)	**1.68 (1.12-2.53)**<br>1.39 (0.96-2.01)<br>1.43 (0.79-2.61)	0.45
Model 3: Model 2 covariates + potential moderators	Premenopausal referent<br>Postmenopausal referent<br>Hysterectomy referent	1.24 (0.99-1.55)<br>0.98 (0.81-1.20)<br>0.94 (0.66-1.35)	1.21 (0.91-1.61)<br>0.99 (0.74-1.32)<br>1.42 (0.92-2.20)	1.45 (0.95-2.20)<br>1.22 (0.84-1.76)<br>1.35 (0.73-2.48)	0.53

Model 2 covariates: age, ethnicity, Townsend deprivation index, alcohol status, daily alcohol intake, days exercised (walked, moderate and vigorous), length of working week, chronotype, job asthma risk and job medical required. Model 3 data are adjusted for Model 2 covariates plus potential moderators sleep duration, sleep medication, smoking status, smoking pack years and BMI.

Supplementary table 21: Adjusted odds (95% CI) of having moderate-severe asthma by current shift work exposure with day workers as referent, stratified by menopause status defined as self-reported or older than 50. (n=124,374)

Premenopausal<br>Postmenopausal<br>Hysterectomy	Current work schedule				Menopause status-shift work interaction
	Day workers	Shift work, but never or rarely night shifts	Irregular shift work including nights	Permanent night shift work	
Total cases (% of total sample size)	605 (1.87%)<br>1,300 (2.05%)<br>291 (2.94%)	80 (2.53%)<br>139 (2.21%)<br>36 (3.00%)	48 (2.29%)<br>64 (2.15%)<br>24 (4.20%)	21 (2.97%)<br>37 (2.93%)<br>12 (4.41%)	
Total sample size	32,406<br>63,516<br>9,910	3,164<br>6,288<br>1,200	2,097<br>2,981<br>572	706<br>1,262<br>272	
Model 1: Age-adjusted	Premenopausal referent<br>Postmenopausal referent<br>Hysterectomy referent	**1.36 (1.08-1.73)**<br>1.09 (0.91-1.30)<br>1.02 (0.72-1.45)	1.24 (0.92-1.66)<br>1.08 (0.84-1.40)<br>1.45 (0.95-2.22)	**1.62 (1.04-2.52)**<br>**1.47 (1.05-2.05)**<br>1.53 (0.85-2.75)	0.48
Model 2: Multivariable-adjusted	Premenopausal referent<br>Postmenopausal referent<br>Hysterectomy referent	**1.36 (1.07-1.72)**<br>1.05 (0.88-1.26)<br>0.98 (0.69-1.40)	1.28 (0.94-1.73)<br>1.11 (0.86-1.43)<br>1.49 (0.97-2.30)	**1.63 (1.04-2.55)**<br>1.40 (1.00-1.97)<br>1.43 (0.79-2.61)	0.47
Model 3: Model 2 covariates + potential moderators	Premenopausal referent<br>Postmenopausal referent<br>Hysterectomy referent	1.26 (0.99-1.61)<br>0.98 (0.82-1.18)<br>0.94 (0.66-1.35)	1.22 (0.90-1.65)<br>1.02 (0.79-1.32)<br>1.42 (0.92-2.20)	1.47 (0.94-2.30)<br>1.19 (0.85-1.69)<br>1.35 (0.73-2.48)	0.55

Model 2 covariates: age, ethnicity, Townsend deprivation index, alcohol status, daily alcohol intake, days exercised (walked, moderate and vigorous), length of working week, chronotype, job asthma risk and job medical required. Model 3 data are adjusted for Model 2 covariates plus potential moderators sleep duration, sleep medication, smoking status, smoking pack years and BMI.

Supplementary table 22: Adjusted odds (95% CI) of having moderate-severe asthma by current shift work exposure with day workers as referent, stratified by menopause status defined as self-reported or older than 50. Participants on HRT or currently taking OCP are excluded (n=86,485)

Premenopausal<br>Postmenopausal<br>Hysterectomy	Current work schedule				Menopause status-shift work interaction
	Day workers	Shift work, but never or rarely night shifts	Irregular shift work including nights	Permanent night shift work	
Total cases (% of total sample size)	531 (1.81%)<br>682 (1.68%)<br>99 (2.61%)	72 (2.50%)<br>69 (1.80%)<br>11 (2.41%)	44 (2.26%)<br>36 (1.91%)<br>8 (3.45%)	17 (2.64%)<br>24 (3.11%)<br>4 (4.30%)	
Total sample size	29,282<br>40,665<br>3,799	2,877<br>3,834<br>457	1,947<br>1,885<br>232	643<br>771<br>93	
Model 1: Age-adjusted	Premenopausal referent	**1.39 (1.08-1.78)**	1.26 (0.92-1.71)	1.48 (0.90-2.41)	0.36
	Postmenopausal referent	1.08 (0.84-1.38)	1.16 (0.83-1.63)	**1.90 (1.25-2.87)**	
	Hysterectomy referent	0.92 (0.49-1.73)	1.33 (0.64-2.76)	1.68 (0.60-4.66)	
Model 2: Multivariable-adjusted	Premenopausal referent	**1.39 (1.08-1.79)**	1.31 (0.96-1.80)	1.51 (0.92-2.47)	0.35
	Postmenopausal referent	1.03 (0.79-1.33)	1.20 (0.85-1.68)	**1.89 (1.24-2.87)**	
	Hysterectomy referent	0.90 (0.48-1.71)	1.47 (0.69-3.10)	1.75 (0.62-4.96)	
Model 3: Model 2 covariates + potential moderators	Premenopausal referent	1.28 (0.99-1.66)	1.26 (0.91-1.73)	1.35 (0.82-2.22)	0.44
	Postmenopausal referent	0.96 (0.74-1.25)	1.10 (0.78-1.56)	**1.57 (1.02-2.42)**	
	Hysterectomy referent	0.86 (0.45-1.64)	1.35 (0.63-2.86)	1.67 (0.58-4.80)	

Model 2 covariates: age, ethnicity, Townsend deprivation index, alcohol status, daily alcohol intake, days exercised (walked, moderate and vigorous), length of working week, chronotype, job asthma risk and job medical required. Model 3 data are adjusted for Model 2 covariates plus potential moderators sleep duration, sleep medication, smoking status, smoking pack years and BMI.

Supplementary table 23: Adjusted odds (95% CI) of having moderate-severe asthma by current shift work exposure with day workers as referent, stratified by sex and menopause status defined as self-reported or older than 50 (n=241,724). Interaction terms consider whether the likelihood of moderate-severe asthma varies differently across shift work frequency categories when comparing males with the three female groups considered separately

Male / Female premenopausal/ Female, postmenopausal/ Female, hysterectomy	Current work schedule				Sex-shift work interaction (sub-cohort + males)
	Day workers	Shift work, but never or rarely night shifts	Irregular shift work including nights	Permanent night shift work	
Total cases (% of total sample size)	1,469 (1.56%)<br>605 (1.87%)<br>1,300 (2.05%)<br>291 (2.94%)	161 (1.62%)<br>80 (2.53%)<br>139 (2.21%)<br>36 (3.00%)	139 (2.38%)<br>48 (2.29%)<br>64 (2.15%)<br>24 (4.20%)	73 (3.13%)<br>21 (2.97%)<br>37 (2.93%)<br>12 (4.41%)	
Total sample size	93,937<br>32,406<br>63,516<br>9,910	9,941<br>3,164<br>6,288<br>1,200	9,709<br>2,097<br>2,981<br>572	3,763<br>706<br>1,262<br>272	
Model 1: Age-adjusted	Male referent	1.05 (0.89-1.23)	0.94 (0.79-1.13)	0.91 (0.69-1.20)	
	Premenopausal referent	**1.36 (1.08-1.73)**	1.24 (0.92-1.66)	**1.62 (1.04-2.52)**	**0.03**
	Postmenopausal referent	1.09 (0.91-1.30)	1.08 (0.84-1.40)	**1.47 (1.05-2.05)**	0.19
	Hysterectomy referent	1.02 (0.72-1.45)	1.45 (0.95-2.22)	1.53 (0.85-2.75)	0.14
Model 2: Multivariable-adjusted	Male referent	1.08 (0.92-1.28)	0.98 (0.82-1.17)	0.95 (0.72-1.26)	
	Premenopausal referent	**1.36 (1.07-1.72)**	1.28 (0.94-1.73)	**1.63 (1.04-2.55)**	**0.03**
	Postmenopausal referent	1.05 (0.88-1.26)	1.11 (0.86-1.43)	1.40 (1.00-1.97)	0.16
	Hysterectomy referent	0.98 (0.69-1.40)	1.49 (0.97-2.30)	1.43 (0.79-2.61)	0.12
Model 3: Model 2 covariates + potential moderators	Male referent	1.05 (0.89-1.24)	0.95 (0.80-1.14)	0.90 (0.68-1.20)	
	Premenopausal referent	1.26 (0.99-1.61)	1.22 (0.90-1.65)	1.47 (0.94-2.30)	**0.04**
	Postmenopausal referent	0.98 (0.82-1.18)	1.02 (0.79-1.32)	1.19 (0.85-1.69)	0.28
	Hysterectomy referent	0.94 (0.66-1.35)	1.42 (0.92-2.20)	1.35 (0.73-2.48)	0.11

Model 2 covariates: age, ethnicity, Townsend deprivation index, alcohol status, daily alcohol intake, days exercised (walked, moderate and vigorous), length of working week, chronotype, job asthma risk and job medical required. Model 3 data are adjusted for Model 2 covariates plus potential moderators sleep duration, sleep medication, smoking status, smoking pack years and BMI.

Supplementary table 24: Adjusted odds (95% CI) of having moderate-severe asthma by current shift work exposure with day workers as referent, stratified by sex and menopause status defined as self-reported or older than 50. Participants on HRT or currently taking OCP are excluded (n=203,835). Interaction terms consider whether the likelihood of moderate-severe asthma varies differently across shift work frequency categories when comparing males with the three female groups considered separately

Male<br>Female premenopausal/<br>Female, postmenopausal/<br>Female, hysterectomy	Current work schedule				Sex-shift work interaction (sub-cohort + males)
	Day workers	Shift work, but never or rarely night shifts	Irregular shift work including nights	Permanent night shift work	
Total cases (% of total sample size)	1,469 (1.56%)<br>531 (1.81%)<br>682 (1.68%)<br>99 (2.61%)	161 (1.62%)<br>72 (2.50%)<br>69 (1.80%)<br>11 (2.41%)	139 (2.38%)<br>44 (2.26%)<br>36 (1.91%)<br>8 (3.45%)	73 (3.13%)<br>17 (2.64%)<br>24 (3.11%)<br>4 (4.30%)	
Total sample size	93,937<br>29,282<br>40,665<br>3,799	9,941<br>2,877<br>3,834<br>457	9,709<br>1,947<br>1,885<br>232	3,763<br>643<br>771<br>93	
Model 1: Age-adjusted	Male referent	1.05 (0.89-1.23)	0.94 (0.79-1.13)	0.91 (0.69-1.20)	
	Premenopausal referent	**1.39 (1.08-1.78)**	1.26 (0.92-1.71)	1.48 (0.90-2.41)	0.06
	Postmenopausal referent	1.08 (0.84-1.38)	1.16 (0.83-1.63)	**1.90 (1.25-2.87)**	**0.04**
	Hysterectomy referent	0.92 (0.49-1.73)	1.33 (0.64-2.76)	1.68 (0.60-4.66)	0.54
Model 2: Multivariable-adjusted	Male referent	1.08 (0.92-1.28)	0.98 (0.82-1.17)	0.95 (0.72-1.26)	
	Premenopausal referent	**1.39 (1.08-1.79)**	1.31 (0.96-1.80)	1.51 (0.92-2.47)	**0.05**
	Postmenopausal referent	1.03 (0.79-1.33)	1.20 (0.85-1.68)	**1.89 (1.24-2.87)**	**0.02**
	Hysterectomy referent	0.90 (0.48-1.71)	1.47 (0.69-3.10)	1.75 (0.62-4.96)	0.50
Model 3: Model 2 covariates + potential moderators	Male referent	1.05 (0.89-1.24)	0.95 (0.80-1.14)	0.90 (0.68-1.20)	
	Premenopausal referent	1.28 (0.99-1.66)	1.26 (0.91-1.73)	1.35 (0.82-2.22)	0.07
	Postmenopausal referent	0.96 (0.74-1.25)	1.10 (0.78-1.56)	**1.57 (1.02-2.42)**	0.06
	Hysterectomy referent	0.86 (0.45-1.64)	1.35 (0.63-2.86)	1.67 (0.58-4.80)	0.52

Model 2 covariates: age, ethnicity, Townsend deprivation index, alcohol status, daily alcohol intake, days exercised (walked, moderate and vigorous), length of working week, chronotype, job asthma risk and job medical required. Model 3 data are adjusted for Model 2 covariates plus potential moderators sleep duration, sleep medication, smoking status, smoking pack years and BMI.

Supplementary table 25: Employment characteristics by current shift work exposure (n=285,140)

	Sex	Day workers	Shift work, but never or rarely night shifts	Irregular shift work including nights	Permanent night shift work
N	F	125,856	12,820	6,793	2,736
	M	109,658	11,606	11,298	4,373
Managers and Senior Officials (%)	F	13.52	11.72	7.64	3.73
	M	24.96	14.89	10.53	5.97
Professional Occupations (%)	F	23.64	9.59	8.01	2.74
	M	28.39	11.01	9.50	4.39
Associate Professional and Technical Occupations (%)	F	17.87	26.95	43.59	38.45
	M	14.46	15.14	20.1	16.33
Administrative and Secretarial Occupations (%)	F	26.11	12.53	4.78	5.81
	M	6.87	5.18	2.97	3.84
Skilled Trades Occupations (%)	F	1.57	2.44	1.44	1.02
	M	12.88	15.85	18.68	13.83
Personal Service Occupations[1] (%)	F	7.91	16.34	25.16	34.25
	M	1.39	5.82	5.85	5.12
Sales and Customer Service Occupations (%)	F	4.47	11.08	3.61	4.46
	M	1.60	4.58	1.25	2.61
Process, Plant and Machine Operatives (%)	F	0.90	1.81	1.78	2.27
	M	5.53	15.59	20.76	25.95
Elementary Occupations[2] (%)	F	4.01	7.53	3.99	7.27
	M	3.91	11.93	10.35	21.95

Data are percentages. [1.] Includes care assistants, nursing staff, childcare staff, animal carers. [2.] Includes farm/forestry/fishing workers, labourers, goods handling.
